# Mapping Molecular Association Networks of Nervous System Diseases via Large-Scale Analysis of Published Research

**DOI:** 10.1371/journal.pone.0067121

**Published:** 2013-06-25

**Authors:** Xiaojun Hu, Dangzhi Zhao, Andreas Strotmann

**Affiliations:** 1 Medical Information Centre, Zhejiang University School of Medicine, Hangzhou, China; 2 School of Library and Information Studies, University of Alberta, Edmonton, Canada; 3 GESIS – Leibniz Institute for the Social Sciences, Cologne, Germany; Innsbruck Medical University, Austria

## Abstract

Network medicine has been applied successfully to elicit the structure of large-scale molecular interaction networks. Its main proponents have claimed that this approach to integrative medical investigation should make it possible to identify functional modules of interacting molecular biological units as well as interactions themselves. This paper takes a significant step in this direction. Based on a large-scale analysis of the nervous system molecular medicine literature, this study analyzes and visualizes the complex structure of associations between diseases on the one hand and all types of molecular substances on the other. From this analysis it then identifies functional co-association groups consisting of several types of molecular substances, each consisting of substances that exhibit a pattern of frequent co-association with similar diseases. These groups in turn exhibit interlinking in a complex pattern, suggesting that such complex interactions between functional molecular modules may play a role in disease etiology. We find that the patterns exhibited by the networks of disease – molecular substance associations studied here correspond well to a number of recently published research results, and that the groups of molecular substances identified by statistical analysis of these networks do appear to be interesting groups of molecular substances that are interconnected in identifiable and interpretable ways. Our results not only demonstrate that networks are a convenient framework to analyze and visualize large-scale, complex relationships among molecular networks and diseases, but may also provide a conceptual basis for bridging gaps in experimental and theoretical knowledge.

## Introduction

Information science can help to identify interesting approaches and provide new perspectives for scientific research [1], [2]. Currently, the concept of network medicine is gaining attention in biomedical research and providing a new promising approach to discovering targets for the treatment of diseases [3]–[8]. Advocates of the network medicine approach foresee in particular its potential to provide an improved view of the whole system of the human body, its diseases and their contributing factors, and to thus help bridge the gap between molecular biology and clinical medicine [3]. However, most attention in this field has so far been directed towards molecular networks such as protein interaction networks, metabolic networks, regulatory networks, and RNA networks [3], [9]. Network medical analyses of the full range of molecular substances and their documented disease associations and attempts to elicit patterns such as molecular functional modules from them are still largely missing.

The molecular basis of a disease is very complex, especially so for the aptly-named complex diseases. For example, there is no ‘cancer gene’. A typical cancer patient has mutations in a few dozen of about 300 genes, an elusive combinatorial problem whose complexity is increasingly a worry to the medical community [10]. Similarly, the genomics field has been plagued by examples in which data have resulted in an unacceptably high rate of false positives [9]. One striking example of this is research that was undertaken to replicate published associations between 85 DNA variants and acute coronary syndromes. Of the 85 variants tested, only 1 showed a rise to a nominally significant *P* value, highlighting a complete lack of support for the validity of hypothesis that any of the variants previously reported in scores of publications are associated with acute coronary syndromes [9], [11].

On the other hand, current research suggests that it is not enough to know a precise list of “disease genes”, but rather that diseases should be viewed as the breakdown of specific functional modules rather than single or small groups of genes, where discernible modules consist of an interlocking network of genes, transcription factors, RNAs, enzymes, and metabolites [12], and where any given molecular entity is in turn usually associated with several diseases [8].

In addition, disease-proteins have been found to exhibit more protein-protein interactions than do non-disease proteins [3]. Therefore, it is useful to identify multiply-associated molecular entities acting on a system’s disease genes, and to reveal their documented interrelationships as integrated over a large set of publications.

Nervous system diseases include many complex diseases and syndromes, which are involved in several systems and cause disorders of activities of human body. Recent research has found that there exist networks between neuro-degenerative diseases [13], suggesting that systems- based approaches are becoming *de rigueur* in identifying breakthrough discoveries in science from the seemingly infinite volumes of data generated using modern technologies [8].

In this study, we experiment with a novel non-invasive, information science-based approach to providing an overview of a wide range of disease-associated molecular substances (rather than focusing on associated proteins only) in nervous system diseases. Based on 28,652 records of nervous system disease research published during the period 1965–2012, we identify and visualize major interrelationships in disease-molecular substance association networks derived from these.

Instead of focusing on biological network itself only, the objectives of this study are as follows:

1. to explore a new approach for mapping networks of disease-gene associated molecular substances in nervous system diseases based on large-scale text-analysis rather than focus on biological networks only, in order to identify major substances that are most strongly or widely associated to clinical diseases and their interrelationships at the molecular level;
2. to reveal networks of diseases associated via their molecular bases, in order to provide a novel integrative perspective for clinical concepts rather than focusing on a single disease as a meta-analysis would do.
3. to construct a bridge for connecting experimental research and clinical medicine in a new integrated perspective, and to present an overview of molecular substances and nervous system diseases in a new pattern to complement traditional review and evidence-based medicine.

## Results

The results provide a series of visualizations of networks of molecular substances associated with nervous system disease genetics, derived and integrated from a large collection of published research (Note that this approach is very different from the traditional meta-analysis one, as the methodology to obtain and visualize these networks explains in the Materials and Method section).

### Diseases and their Highly Associated Molecular Substances

**Figure 1:**
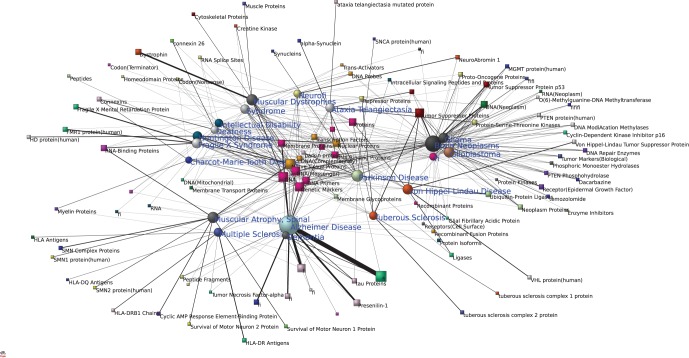
Diseases, associated major molecular substances and their interrelationships. Circular nodes denote diseases, square nodes denote molecular substances. Nodes in the center of the network indicate that they play major roles in that these substances are associated with most of the diseases. Nodes in the periphery of network indicate that they play more minor roles in the network and more specific roles in specific diseases. The width of a line that connects a square node with a circular node is proportional to the weight of this link, as is its grey-scale value, with wider and darker lines signifying higher link weights.

Fig. 1 visualizes major connections in the disease - molecular substance association network of nervous system diseases. 20 nervous system diseases and 100 molecular substances highly associated to them (in short: major molecular substances) are displayed according to the structure of this network. Based on network characteristics and structure, the more extensive the associations of a node to other ones in the network, the larger that node is visualized; the more closely associated two nodes are with each other biologically, the closer they are visualized [3], [9]. In addition, the color of a node (available only in the electronic version of the present paper) indicates the number of links to a node.

The visualization identifies different subnetworks, each consisting of a disease (circular node) and its associated substances (square nodes). Alzheimer disease is the largest disease node in the network, brain neoplasms are second, and Parkinson’s disease is third, indicating that they are most extensively associated to molecular substances in their respective subnetworks, which suggests that they are the three most complex of the system diseases in this network.

One can identify the closely associated major molecular substances of a disease by the sizes and distances of nodes in each subnetwork. In the Alzheimer disease subnetwork, for example, the apolipoproteins E node is clearly largest, with the closest association to the Alzheimer disease node. This is consistent with published research findings that apolipoproteins E play a crucial role in this disease [14]. Furthermore, we can see that apolipoprotein E4, membrane proteins, amyloid beta-protein precursor, amyloid beta-peptides, presenilin-1, tau proteins, and peptide fragments, are all closely connected to Alzheimer disease. Again, these findings are supported by other research [13], [15], [16].

In addition, one can identify closely related diseases by the distance between their nodes. Multiple sclerosis, spinal muscular atrophy, and dementia nodes are close to Alzheimer disease, suggesting that the molecular substances involved in these diseases are highly associated biologically.

Another high density subnetwork that we can identify in Fig. 1 is that of brain neoplasms and associated molecular substances. Tumor suppressor proteins, neoplasms DNA, proto-oncogene proteins, tumor suppressor protein p53, messenger RNA, neoplasm proteins, epidermal growth factor receptor, protein-serine-threonine kinases, DNA repair enzymes, MGMT protein, DNA modification methylases, DNA-binding proteins, and biological tumor markers, are all highly associated with brain neoplasms, reflecting that they play important roles in brain tumors. These results, too, are consistent with current findings [17]–[19]. Cancer types glioma, glioblastoma, astrocytoma are closely similar to brain neoplasms in the sense of associated molecular substances. Von Hippel-Landau disease, associated with increased risk of tumours, is more loosely connected to this subnetwork.

Parkinson disease is a complex disease, which involves complex networks of molecular bases [20]. As the result shows (Fig. 1), ubiquitin-protein ligases, parkin protein, nerve tissue proteins, alpha-synuclein, synucleins, protein-serine-threonine kinases, SNCA protein, ligases, are closely associated with the disease, which again is supported by recent findings [21]–[23].

Besides helping to identify disease-associated major molecular substances, in the center of the network (Fig. 1) one may find some generic molecular substances connecting to many diseases, suggesting that they play “common” roles in the molecular basis of diseases. However, this is also true of some quite specific substances, where a single molecular substance is associated with several diseases. Take tumor suppressor proteins, for example, which not only connect to brain neoplasms, but also to von Hippel-Lindau disease, ataxia telangiectasia, tuberous sclerosis, and glioblastoma, revealing an interrelationship between these diseases – in this case, an associated increased risk of developing tumors.

### Major Molecular Substances and Associated Diseases

We determined 15 special proteins that are highly associated with nervous system diseases, each of them related to at least 20 diseases (Table 1). Clearly, the result suggests that it is difficult to consider diseases as being consistently independent of one another at the molecular level. However, what stories do their patterns about the interrelationship among diseases at the molecular level? How can we find the “hubs” of molecular substances in an integrated view?

**Table 1:** 15 proteins and the number of nervous system diseases they are associated with.

Protein	NAS*	Protein	NAS*
Tumor Suppressor Protein p53	38	tau Proteins	27
Glial Fibrillary Acidic Protein	37	Dystrophin	25
Histones	37	Presenilin-1	23
Apolipoprotein E4	32	HD protein, human	22
FMR1 protein, human	29	Cyclic AMP Response Element-Binding Protein	20
Fragile X Mental Retardation Protein	28	Tuberous sclerosis complex 2 protein	20
Proto-Oncogene Proteins c-bcl-2	28	Methyl-CpG-Binding Protein 2	20
Amyloid beta-Protein Precursor	27		

* NAS: number of associated diseases.

**Figure 2:**
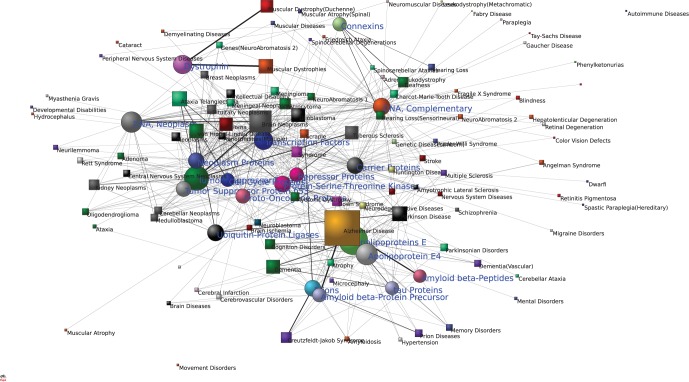
Major molecular substances, associated diseases and their interrelationships. Circular nodes denote molecular substances, square nodes denote diseases. Several high density “node groups” are present in the integrated network. Nodes in the network periphery indicate that they with loose connected to other nodes in the network. The color of a square node indicates the number of circular nodes that this square node links to sufficiently.

Fig. 2 displays the 20 most highly associated molecular substances (circular nodes) involved in nervous system diseases (square nodes) and their interrelationships according to network connectivities (page limitations restrict us to showing only 20 major molecular substances). To focus on uncovering interrelationships among diseases, we ignore the generic substances described above, as they tend to dominate the connectivity in these networks and add “noise” to the data. The visualization facilitates an integrated understanding of diseases.

In contrast to Fig. 1, we see an integrative, high density network emerging in Fig. 2. One can easily “judge” the “position” of these major molecular substances associated with nervous system diseases according to the size of their nodes and their locations in the networks. Clearly, the apolipoproteins E node stands out, being connected to more than ten diseases such as Alzheimer disease, dementia, cognition disorders, vascular dementia, Parkinson disease, multiple sclerosis, Down syndrome, atrophy, cerebrovascular disorders, memory disorders.

We notice an interesting phenomenon in Fig. 2. Several densely clustered node groups are present in the integrated network. They show, not simply clusters of molecular substances, but clusters of diseases and associated substances, such as, a cluster of “apolipoproteins E” associated diseases and substances, and a “tumor suppressor proteins” associated one.

Around “apolipoproteins E”, one can find apolipoprotein E4, amyloid beta-protein precursor, amyloid beta-peptides, tau proteins, prions and their associated diseases: most of the molecular substances in this group are highly related to diseases to which apolipoproteins E are associated (described above). We also see that the disease most closely associated to prions is Creutzfeldt-Jakob syndrome, consistent with previous findings [13].

Clearly, one can identify the “tumor suppressor proteins” associated cluster as the largest and densest subnetwork in this figure, in close vicinity to associated diseases and other major molecular substances. This shows neoplasm DNA, neoplasm proteins, tumor suppressor protein p53, proto-oncogene proteins, highly interrelated with brain neoplasms, tuberous sclerosis, von Hippel-Lindau disease, ataxia telangiectasia, glioblastoma, glioma, astrocytoma, cerebellar neoplasms, neurofibromatosis1, Down syndrome.

As the top-left of Fig. 2 shows, the highly associated diseases of dystrophin are muscular dystrophies and Duchenne muscular dystrophy, while connexins are close related with deafness, sensorineural hearing loss, charcot-marie-tooth disease, and hearing loss.

In addition, we see some groups such as “transcription factors”, “complementary DNA”, “carrier proteins”, “ubiquitin-protein ligases”, “protein-serine-threonine kinases” displayed in the center of network, each of them connected to many nodes belonging to different clusters, indicating that they act as important “mediators” for different diseases.

One may also find kidney neoplasms (at the middle left of Fig. 2) and breast neoplasms (at the top-left) appearing close to the associated group of “tumor suppressor proteins”, which reflects that non-nervous system diseases also have some interrelationships to nervous system diseases.

Clearly, the visualized results provide us a good understanding of interrelationships between major molecular substances and their associated diseases in an integrating view. In particular, they suggest that different functional disease modules can overlap [3], [9].

### Clusters of Major Molecular Substances in Nervous System Diseases

**Figure 3:**
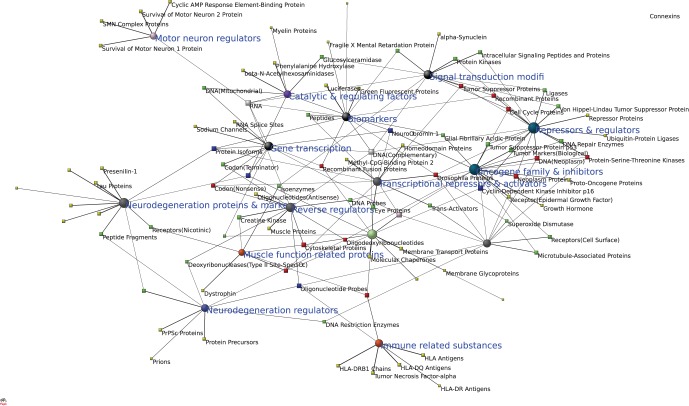
Clusters of major molecular substances genetically associated with nervous system diseases. Circular nodes represent clusters of substances frequently associated to similar diseases, and square nodes denote molecular substances. The size of a circular node corresponds to the sum of substances in the cluster.

To better identify and label major molecular substances which play the role of “hubs” in the huge network of molecular entities that are involved in nervous system diseases, we remove some generic substances which appear common to most diseases, and focus on 93 major molecular substances (covering 43 proteins) with nodes with high interconnectivity in the network, to extract 15 clusters by factor analysis, see Table 2 and Fig. 3.

**Table 2:** Factor labels, sizes, and highest loadings–analysis of 93 major associated molecular substances.

Factor	Size	High loading
Oncogene family and inhibitors	13	0.874
Transcription	9	0.726
Neurodegeneration proteins & markers	11	0.911
Immune related substances	6	0.967
Reverse regulators	11	0.931
Repressors & regulators	11	0.844
Motor neuron regulators	5	0.892
Catalytic & regulated factors	8	0.814
Transcriptional repressors & activators	6	0.802
Neurodegeneration regulators	7	0.814
Muscle function related proteins	6	0.644
Biomarkers	6	0.861
Signal transduction modifiers	7	0.835
Microtubule regulators	4	0.658
Transporters & assistors	8	0.960

Fig. 3 displays co-association groups of major molecular substances in nervous system diseases – factors of molecules that tend to be associated to the same diseases. In this figure, factors and molecular substances are present as circular and square nodes, resp., the rendered size of a factor node is accumulated from loadings (both primary and secondary) in the structure matrix. In addition, the color of a molecular substance node (available only in the electronic version of the present paper) indicates the number of factors on which this molecule loads.

We use the term “co-association group” here in a cautious sense, as the functions of a molecular substance are very complex in the biological process, but we do label each factor according to its perceived biological function. We find that each co-association group generates a brief story about actions on biological processes involved in nervous system diseases when examining known roles of its member substances. The following outlines the perceived function of each factor in turn.

#### Oncogene family and inhibitors

As shown in Fig. 3, this co-association group of molecular substances plays an important role in the biological processes of cancers. Mutations that lead to epidermal growth factor receptor over-expression (known as upregulation) or over-activity have been associated with a number of cancers. Growth hormone may have bidirectional impacts on tumor growth potential [24].

#### Transcription

This co-association group of molecular substances plays vital roles in gene transcription (Fig. 3). It is well known that RNA splice sites and codon terminators are instrumental in transcription to RNA. Recent research indicates that alternative splicing is particularly involved in regulation of neuronal sodium channel molecule transcription [25].

#### Neurodegeneration proteins & markers

This co-association group plays significant roles in the biological process of neurodegeneration. The substances often serves as biomarkers (Fig. 3), i.e., they are measured and evaluated as indicators of normal biological processes vs. pathogenic processes. Pau proteins are involved in both physiological and pathological conditions: in Alzheimer's disease a proportion of tau protein becomes abnormally phosphorylated and is no longer associated with axonal microtubules but instead accumulates in paired helical filaments throughout affected nerve cells [26]. Mutations of presenilin 1 or amyloid precursor protein cause familial susceptibility to Alzheimer's disease [27].

#### Immune related substances

This group of molecular substances is associated with immune response (Fig. 3). The primary role of tumor necrosis factor-alpha is in the regulation of immune cells.

#### Reverse regulators

This group of molecular substances is associated with reversible regulations in the biological process of nervous system diseases (Fig. 3). Elevation of creatine kinase is an indication of damage to muscle. Antisense oligonucleotide acting on specific mRNA can inhibit its expression and then induce a blockade in the transfer of genetic information from DNA to protein [28].

#### Repressors & regulators

This group of molecular substances is associated with regulating the biological process of diseases (Fig. 3). The Von Hippel–Lindau tumor suppressor, also known as pVHL, is a protein that in humans is encoded by the VHL gene. Mutations of the VHL gene are associated with Von Hippel–Lindau disease. The protein encoded by this gene is a component of the protein complex that possesses ubiquitin ligase E3 activity. Repressor proteins prevent RNA polymerase from creating messenger RNA.

#### Motor neuron regulators

This associated group of molecular substances is important for the maintenance of specialized nerve cells called motor neurons (Fig. 3). Cyclic AMP response element-binding protein prevents endothelial permeability increase [29].

#### Metabolism

This group of molecular substances is associated with catalysis and regulation for metabolism and energy (Fig. 3). Mutations in mitochondrial DNA, most of which codes for the core ATP energy metabolism, mutations in genes coding for proteins responsible for transport of ATP from the mitochondrium to the cell, and mutations in the gene coding for phenylalanine hydroxylase all lead to severe metabolic disorders.

#### Transcriptional repressors & activators

This group of molecular substances acts as a transcriptional repressors and activators (Fig. 3). However, the idea that methyl-CpG-binding protein 2 functions as an activator is relatively new and remains controversial [30]. Most of the time, homeodomain proteins act in the promoter region of their target genes as complexes with other transcription factors.

#### Neurodegeneration regulators

The substances in this group are closely associated with proteins implicated in neurodegeneration Direct inhibition of prion protein function by PrP(Sc) may be necessary for neurodegeneration in prion disease [31].

#### Muscle function related proteins

This associated group of molecular substances is related to muscle function (Fig. 3). Functional deactivation related to calcium channels is associated with myodystrophy.

#### Biomarkers

This group of molecular substances is associated with bioluminescence and can be used as markers for particular characteristics (Fig. 3).

#### Signal transduction modifiers

This group of molecular substances is associated with modifying the transmission of molecular signals from a cell's exterior to its interior (Fig. 3). Protein kinases are known to regulate the majority of cellular pathways, especially those involved in signal transduction.

#### Microtubule regulators

This co-association group of molecular substances make up microtubules or regulate their stability (Fig. 3). Microtubule-associated proteins have been found to carry out a wide range of functions, including both stabilizing and destabilizing microtubules, guiding microtubules towards specific cellular locations, cross-linking microtubules and mediating the interactions of microtubules with other proteins in the cell [32]. One form of superoxide dismutase is present in mitochondria and peroxisomes.

#### Transporters & assistors

This group of molecular substances is associated with transport proteins (Fig. 3). Membrane transport proteins, functioning either as channels or transporters, are the gatekeepers that control contact with the world outside the cell by catalyzing the flow of ions and molecules across cell membranes. Malfunctioning transport proteins can lead to cancer, inflammatory, and neurological diseases [33]. Some types of molecular chaperones are involved in transport across membranes [34].

### Molecular Substances Discovered in the Recent Decade

As the biological process of nervous system diseases is very complex and the function of many molecular substances is still uncertain, newly discovered disease gene associated molecular substances need to be paid more attention to even if they do not meet the threshold of interconnectedness for the integrated networks shown as Figs. 1–3 A total of 308 molecular substances that emerged during 2001–2012 were determined in our study, and 12 of these new molecular substances were identified with rich associations to 43 out of 93 major molecular substances which we determined in the previous section.

**Figure 4:**
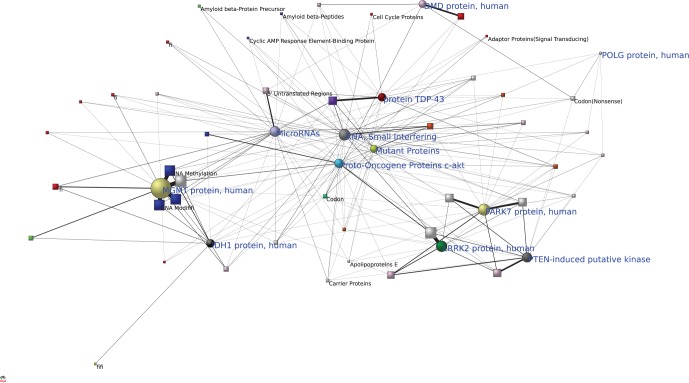
Interrelationships between some recently discovered substances and major molecular substances. Recent substances are displayed as circular nodes, major molecular substances as square nodes. Nodes in the centre indicate “general” functions. The distance between two nodes indicate the strength of their biological association.

We recall that one of networks properties is that functionally related components are likely to be found in their network-based vicinity [3], [9]. In this sense, the visualization of the results shown in Fig. 4 may help us to uncover some potential functions of newly discovered substances by their interrelationships with the major substances whose functions are well understood. As Fig. 4 shows, LRRK2 protein, PTEN-induced putative kinase, PARK7 protein, are close to protein-serine-threonine kinases, ubiquitin-protein ligases, protein kinases, intracellular signaling peptides and proteins, and oncogene proteins, indicating that they are closely related to them biologically. In this way, one can find protein TDP-43, mutant proteins, small interfering RNA, are close to DNA-binding proteins and nuclear proteins, microRNAs related to 3′ untranslated regions, DMD protein near to dystrophin, MGMT protein and IDH1 protein quite close to a group of major substances acting as regulators and markers in the biological process of cancer. Moreover, we find that, small interfering RNA, microRNAs, mutant proteins, and proto-oncogene proteins c-akt, are associated to several major substances, indicating that they may have “general” functions acting on molecular basis of nervous system diseases.

### Risk of Bias within Studies

The purpose of present study is to explore a new way to study widely agreed-upon interrelationships between diseases at the molecular level to help bridge the gaps between experimental research and clinical concepts. This consensus is discovered through a census of nearly 30,000 research journal publications as to which links they describe most frequently. Infrequently discussed connections, many of which presumably correspond to “discoveries” that did not pan out, are effectively ignored in the figures that we present as results.

This approach, while common in network medicine, is in stark contrast to traditional meta-analysis, which focuses on the optimal evidence for clinical protocols for a single diseases by evaluating a small number of studies, from which irreproducible ones are weeded out one by one. This manual process of separating useful from useless information is usually documented as PRISMA (Preferred Reporting Items for Systematic Reviews and Meta-Analyses) and is required when publishing systematic reviews and meta-analyses [35] to help estimate biases.

The issue of bias has not really been considered in network medicine as well as in literature data mining for biology yet [3], [36], and a PRISMA equivalent for network medicine studies such as ours is yet to be defined. There are good reasons for this, and not so good ones. Immaturity of the network medicine field with a resulting lack of consensus on identifying and reporting bias is one main reason, though not a good one. The fact that network medicine studies only extract, study, and discuss features that enjoy a broad consensus is a good reason why PRISMA-style reporting is largely useless here, as no intellectual weeding of the literature takes place, even if weeding out spurious reports (“noise”) is one of the side effects in practice of concentrating on broad consensus features (“signal”).

Nevertheless, we do acknowledge that there is some risk of biases within this study which could influence the validation of results.

1. All study types of 28562 records are included in the data analysis, which would lead to selection bias;
2. All data of 28562 records are collected from the PubMed database, which would lead to information bias.

## Discussion

In this study, we apply a network analysis based approach originally developed in information science to a comprehensive set of publications touching the genetics of neural system diseases to identify major molecular substances in an integrated network of associations derived from the literature.

Our results demonstrate that it is problematic to consider diseases as being consistently independent of one another, and instead reflect “resonating perturbations” in networks of associated molecular substances and their interrelationships in the disease-molecular substance complex (Figs. 1–2). Specifically, 20 nervous system diseases and their associations with more than 100 major molecular substances were studied, revealing that 20 molecular substances highly associated with nervous system diseases, each of them connected both to a number of diseases and all of them interrelated with each other, among them 15 particular proteins that are highly associated with nervous system diseases, each connected to at least 20 diseases, findings that are consistent with previous hypothesis of the human diseasome [3], [37].

Remarkably, our network-based analysis reveals functional co-association groups of major molecular substances, such that each co-association group generates a story of its member substances involved in biological processes which are critical in nervous system diseases.

First, we find large-scale literature-based evidence that networks of substances operating in biomedical systems are not random, but exhibit interesting structural features, as exhibited in Figs. 1–4. Generic substances whose nodes connect to most diseases’ nodes appear in the center of these networks, whereas the more specifically disease-connected molecular nodes appear in the periphery (Fig. 1).

Second, we show that the 20 molecular substances most highly associated to nervous system diseases are closely linked to two large clusters centered on “brain neoplasms” and “Alzheimer disease”, exhibiting high density interrelationships within each group. 15 proteins are particularly highly associated with nervous system diseases, each of them connected to at least 20 diseases (Table 1). This pattern appears when removing the generic substances (Fig. 2).

Third, we reveal that there exist functional co-association groups in major molecular substances, each group generating an interesting narrative about biological processes involved in nervous system diseases. In summary, our network-based results indicate fundamental frameworks of major molecular substances that are highly associated with nervous system diseases and their interrelationships, derived from integration of a huge literature survey, standing out from a “daunting” number of molecular entities [3], [9].

The key findings of our study are that not only revealing functional co-association groups exist in the network of major molecular substances involved in nervous system diseases, but also that the interrelationships within each group and among different groups can be identified. As Fig. 3 shows, nodes involved in a certain function aggregate in a co-association group, and groups with similar or related function are shown in close proximity to one another in such a network. Groups of molecular substances with “generic” functions or “essential” roles in the biological process involved in nervous system diseases, such as the “association groups” of gene transcription, reverse regulators, transcriptional repressors & activators, transporters & assistors, appear at the center of networks.

From these results we can see that the identification of network properties present in biomedical systems can provides a significant basis for studying network medicine in the future, which may help to find valid systemic interpretations of the biological mechanism of human diseases.

As the functions of most newly discovered molecular substances are uncertain, and biological processes of many nervous system diseases are very complex and still unclear [8], [9], the visualization of interrelationships between newly discovered molecular substances and major molecular substances whose functions are well understood provides clues as to the function of the newly discovered molecular substances as well as to an understanding of their biological mechanisms in association to nervous system diseases.

In conclusion, our study visually reveals a fundamental large-scale consensus framework of major molecular substances associated with nervous system diseases and their interrelationships among one another, suggesting that network-based analysis is a convenient approach to analyzing and visualizing large-scale, complex relationships among molecular substances and diseases. Compared to the model of traditional review and evidence-based medicine, our findings not only have profound implications for improved understanding of disease-molecular substance-association networks, but may also provide a conceptual basis for bridging gaps in experimental and theoretical knowledge, in a new integrated perspective.

### The Limitations of this Study

Here we merely explore a new approach for mapping networks of disease-gene associated molecular substances in nervous system diseases based on large-scale text-analysis. This is necessarily a preliminary study, as network medicine is currently still an emerging field, if a promising one for informing both basic research and clinical medicine in the future. Although there are no similar criteria like PRISMA [35] in network medicine [3], we will attempt to address some limitations of our study here.

1. As a first step in the direction of network medicine, the results of our study is preliminary. It now merely sketches a broad framework of molecular associations in a network of nervous system diseases rather than providing precise scientific conclusion on these interrelationships.
2. The statistical analysis in this study is not multiple-dimensional, which may limit the ability of our study to produce deeper findings.
3. We use PubMed database as the only data source to research, and all study types of 28562 records are included in our research, which could lead to information bias and selection bias in this study. To balance this, only items and connections which frequently occur in these records are extracted for analysis, thus presumably focusing on those for which a broad consensus exists in the scientific literature.

## Materials and Methods

### Hypotheses

The study is conducted under the following hypotheses:

1. Each record in PubMed corresponds to a paper which was assessed by reviewers before publication. As such, it represents at least a unit of knowledge about scientific phenomena resulting from research;
2. In scientific fields, the more important the relation between two topics, the more frequently studies deal with them, and the more journal articles are written that include both topics. If there are plenty of articles that discuss similar conclusions on a topic, then these conclusions are acceptably reliable.
3. In nervous system diseases, the more complex biological processes a molecular substance is involved in, the more frequently other substances will be connected with it, and therefore the more articles will discuss such a connection. Therefore, we can construct meaningful networks from frequently documented connections and visualize their interrelationships in the area of nervous system diseases, based on a high threshold of the number of articles on the same kind of findings.

### Data Collection

We used PubMed to collect data for the present study. PubMed is a bibliographical database known for its excellent coverage and indexing of journal publications in the biomedical research fields [6], [38]. We retrieved a total of 28,652 records from PubMed for the years 1965–2012 using “nervous system diseases” and “genes” as MeSH (Medical Subject Headings) terms, and “genetics” as a qualifier for “nervous system diseases”. The year 1965 was chosen because the term “nervous system diseases” was first introduced into MeSH in that year. The actual retrieval was carried out on Apr.12, 2012, when complete records of retrieved results were downloaded in XML format.

### Data Analysis

These XML records (our dataset) were then processed by a computer program we developed in order to produce the data we needed. Specifically, we ranked MeSH terms (Descriptors) and Chemicals (Substances) by the number of times they appeared in our dataset, and took the top 1000 from each ranking. We then manually examined the most commonly used descriptors and substances in order to extract the data that can help address our research questions.

Substances are particular molecular entities that are registered in the CAS (Chemical Abstracts Service) Database, including drugs, proteins, and enzymes. Descriptors are terms that describe various facets of biomedical research, including diseases, topics, chromosomes, and substances that have been introduced into MeSH. In order to focus on the interrelationships between major diseases and molecular substances, we first removed chemical drugs for nervous system diseases [39] from the top 1000 chemicals/substances list, and identified the descriptors that represent diseases in the list of descriptors.

We then took the top 100 substances and top 100 diseases, and calculated two co-occurrence matrices for them: a disease – substance matrix, and a substance – substance matrix. A number in the former for disease x and substance y, for example, is the number of articles in which both x and y appear, i.e., articles that are indexed in PubMed using both x and y, indicating the degree of association between x and y as collectively perceived by the indexers based on the content of the articles indexed. We also calculated a year – substance matrix, recording how many articles contain each of the top 100 substances in each year (The flow chart see Fig. S1).

From these three matrices, we extracted four sub-networks for further analyses: (a) a 20×100 disease – substance network that focuses on the top 20 major diseases and represents how these diseases are related to each other and how they relate to the top 100 molecular substances; (b) a 20×100 substance – disease network that focuses on the top 20 molecular substances and represents how these substances are related to each other and how they relate to the top 100 major diseases; (c) a 93×93 substance – substance network that represents the interrelationships among the top 93 molecular substances resulting from removing from the top 100 the “generic substances” (e.g., DNA) that are associated with almost all diseases; and (d) a 12×43 substance – substance matrix that represents the interrelationships between 12 recently discovered substances and the major substances that are related to them. The 12 “new” substances were chosen from the substances that emerged during the years 2001 and 2012, based on whether they have been studied sufficiently. Their 43 related substances are those among the top 100 substances that co-occurred with them in our dataset.

These four networks were then visualized using techniques introduced in previous studies [40]–[42] to aid interpretation as explained in detail below, directly for all the networks except network (c) for which results from the Factor Analysis of the matrix were visualized.

The factor analysis of network (c), i.e., the 93×93 major substance co-occurrence matrix, was performed using SPSS’ Factor Analysis routine in order to reveal the underlying structure of the interrelationships among these substances. Factors were extracted by Principal Component Analysis (PCA), and we took a 15-factor model after exploring different factor models balancing their explanatory powers with their statistical features and model fits including the Scree plot, total variance explained, communalities – how well a variable (i.e., a substance here) is explained by the factor model, and correlation residuals –, the differences between observed correlations and correlations implied by the factor model [43]. This 15-factor model had a good model fit as it explained 66.5% of the total variance, and the differences between observed and implied correlations were smaller than 0.05 for the most part (83%). About 67% of the communalities were above 0.6, with the highest being 0.92.

We applied an oblique rotation (SPSS Direct OBLIMIN) to the factor model, which resulted in a pattern and a structure matrix. As explained in previous studies [40]–[42], we used the pattern matrix to label the factors upon examining the articles containing the substances in the corresponding factors, and used the structure matrix to create the two-dimensional map (Fig. 3).

### Network Visualization

Pajek is one of the most popular network tools in social network analysis and with excellent functions and widely applications for information science [38], [40]–[42], [44]. Compared with designed for biological data text mining in PubMed such as Chilibot, PESCADOR, iHOP [45]–[48], it provides excellent functionality with a wide range of applications in information science [37], [39]–[41]. We use Pajek to visualize the four sub-networks to create the two dimensional maps (Figs. 1–4). In these bipartite graphs, the nodes in the larger partition are represented by squares and the nodes in the smaller partition by circles. For example, in network (a), the 20 diseases are represented on the map as circular nodes and the 100 substances as square nodes; and in network (c), i.e., the network “substance – factor of substances”, the 15 factors are represented as circles and the 93 substances as squares. The layout of these maps is an automatically generated Kamada-Kawai graph layout using link weights (i.e., co-occurrences or factor loadings) as similarity measures between nodes of the two partitions, produced by Pajek [44].

The size of a circular node corresponds to the sum of the weights of links that are sufficiently strong (e.g., with a value of 0.3 or higher in the case of factor loadings). The width of a line that connects a square node with a circular node is proportional to the weight of this link, as is its grey-scale value, with wider and darker lines signifying higher link weights. The color of a square node indicates the number of circular nodes that this square node links to sufficiently: yellow for squares that only link sufficiently to a single circular node, green for those that link to two circular nodes, red for three, and blue for four.

## Acknowledgments

The authors would like to thank Zhu Lijun at the Institute of Neuroscience of the Zhejiang University China for her help with the interpretational labeling of the factor groupings of molecular substances, and Guo Gencheng at the University of Alberta for writing the computer program we used in data processing. This study was carried out during Hu’s visit at the University of Alberta in collaboration with Zhao and Strotmann.

## Supporting Information

Figure S1 **Flow chart depicting data processing of co-occurrence matrices construction.**
(TIF)

Table S1 **Items included in our study vs. PRISMA 2009 Checklist.**
(DOC)
