# Supplementary figures and images for: Mapping Molecular Association Networks of Nervous System Diseases via Large-Scale Analysis of Published Research
Source: PLoS One. 2013 Jun 25;8(6):e67121. doi: 10.1371/journal.pone.0067121 (PMC3692415; doi:10.1371/journal.pone.0067121)

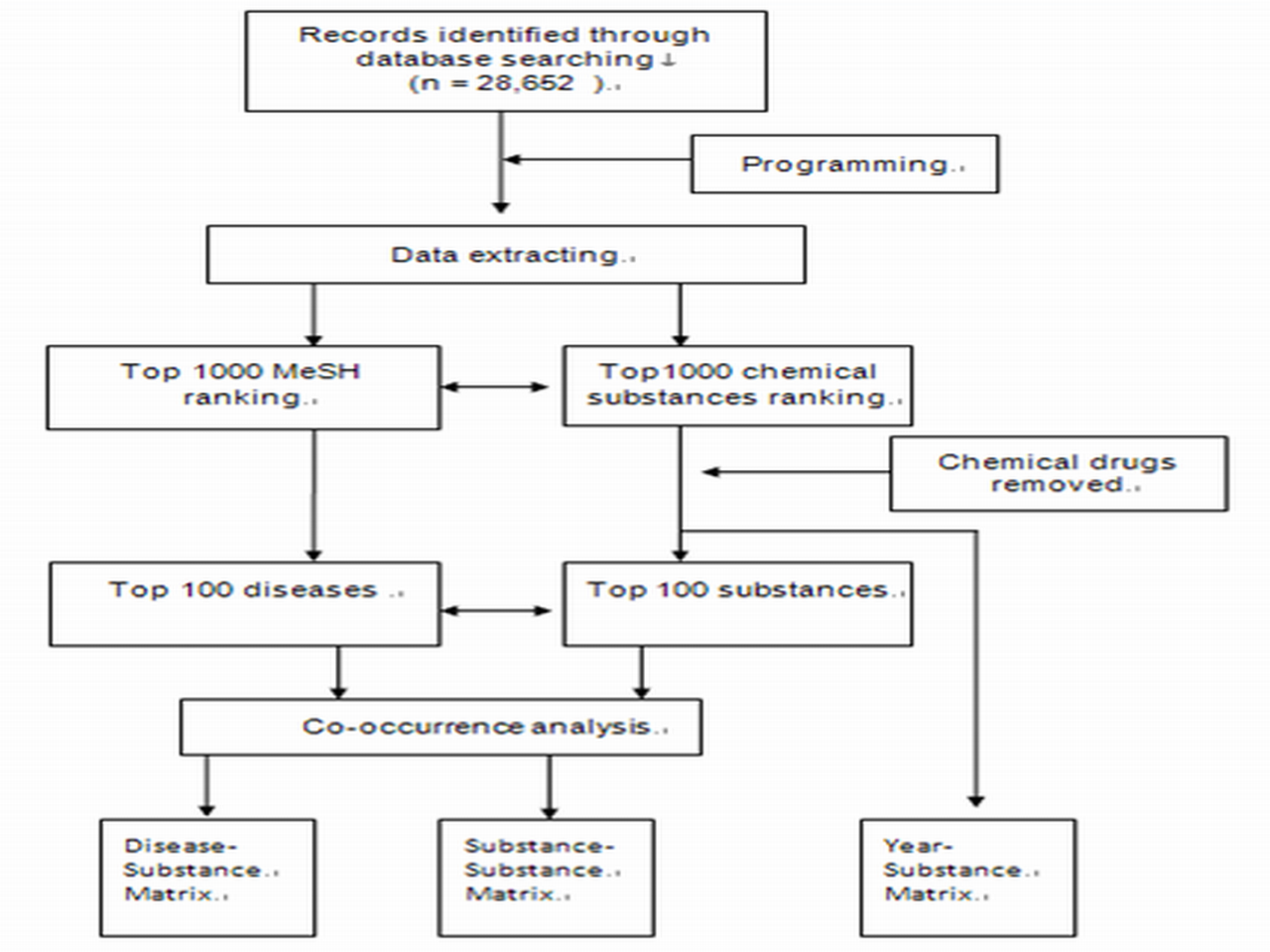

Supplement: Figure S1 — Flow chart depicting data processing of co-occurrence matrices construction. (TIF) [file pone.0067121.s001.tif]
